# Males migrate farther than females in a differential migrant: an examination of the fasting endurance hypothesis

**DOI:** 10.1098/rsos.140346

**Published:** 2014-12-17

**Authors:** Elizabeth A. Gow, Karen L. Wiebe

**Affiliations:** Department of Biology, University of Saskatchewan, 112 Science Place, Saskatoon, Saskatchewan, Canada S7N 5E2

**Keywords:** northern flicker, migration patterns, migratory connectivity, sex differences, differential migration

## Abstract

Patterns of migration including connectivity between breeding and non-breeding populations and intraspecific variation in the distance travelled are important to study because they can affect individual fitness and population dynamics. Using data from 182 band recoveries across North America and 17 light-level geolocators, we examined the migration patterns of the northern flicker (*Colaptes auratus*), a migratory woodpecker. This species is unusual among birds because males invest more in parental care than females. Breeding latitude was positively correlated to migration distance because populations in the north appeared to travel farther distances than southern populations to find wintering locations with little snow cover. Connectivity was strong for populations west and east of the Continental Divide. Contrary to the three main hypotheses for intraspecific variation in migration distance, females wintered, on average, farther north than males, although there was overlap throughout their non-breeding range. This pattern contradicts those of other species found to date and is most consistent with the fasting endurance hypothesis if investment in parental care depletes the energy reserves of male flickers more than females. We thus propose a new factor, parental effort, which may influence optimal wintering areas and migration strategies within birds, and encourage future experimental studies to test the relationship between parental care roles and migration strategies of the sexes.

## 2. Introduction

Birds exhibit diverse strategies for migrating between their breeding and wintering sites, such as variation in routes, timing and distances travelled. Patterns of migration can be described at different taxonomic and spatial scales and vary between species, between populations and also within populations [1]. Many individuals in the Northern Hemisphere show roughly north–south parallel movements meaning that western-breeding populations winter farther west than eastern-breeding populations, on a continental scale [2–5]. However, cases where eastern and western populations cross-over during migration are also known [6]. Populations may also differ in their propensity to migrate based on latitude. For example, northern populations of European robins (*Erithacus rubecula*) are migratory, whereas southern populations are resident [7]. In other cases ‘leapfrog’ migrations may occur where northern-breeding populations winter at more southerly latitudes than do the southern-breeding populations [8]. Chain migration, in contrast to ‘leapfrog’ migration, occurs when winter populations occur in the same north–south sequence as breeding populations [1].

Knowledge about how different populations overlap on the breeding and wintering grounds (migratory connectivity) is important in addressing issues of conservation. For example, when most members of a given breeding population winter in the same location and do not overlap with other populations (strong connectivity), the impacts of harmful environmental effects on the non-breeding grounds, such as habitat disturbance, will be focused on the specific populations [9,10]. However, when members of a breeding population winter in multiple locations and overlap with members of other breeding populations, any negative impact at a certain wintering area is predicted to be spread evenly across the breeding range [10,11]. Many species show intermediate connectivity [10]. Determining the degree of connectivity and the location of wintering areas for different bird species with the aid of technologies such as stable isotopes, genetics and geolocators has been a focus of avian ecology in the last two decades.

At the smaller scale within populations, the propensity to migrate may vary between individuals. Partial migration occurs if some individuals reside on the breeding site year round, while others migrate, whereas differential migration occurs when individuals from a population migrate different distances [1]. With partial and differential migration, wintering areas or migration routes are often based on the sex or age of the individual [12,13]. Chapman *et al*. [14] reviewed eight hypotheses which explain why some individuals migrate farther distances than others, but three hypotheses have received most attention. The arrival time hypothesis [12,15] suggests that one sex (usually the male) benefits more from earlier arrival on the breeding grounds to establish a territory and hence will winter farther north. The body size hypothesis [12] suggests that the larger sex (usually the male) can withstand harsh winter conditions better and hence makes the same prediction: males should winter farther north than females. Likewise, the dominance hypothesis [12] suggests that socially dominant individuals (usually older and male) force subordinate individuals to migrate farther distances.

Rarely discussed, the fasting endurance hypothesis [14] proposes that individuals at a greater risk of starvation migrate farther distances south in order to winter in more benign habitats. Here, we propose a revision to the fasting endurance hypothesis that if parental care is energetically costly, then providing parental care drains energy reserves so that the sex which provides more care should be more willing to travel to a favourable wintering habitat in order to recoup body condition. Thus, we suggest that the pattern most commonly observed in birds, where females migrate farther than males, may be partly explained by sex-related patterns of parental care. In most species, females provide more care than males and so predictions of this hypothesis would usually be the same as the previous three hypotheses: males should be less willing to migrate than females. However, species with reversed sex roles provide a unique opportunity to test how reproductive effort may influence migration strategies because they make the opposite prediction, namely that females would be more likely to spend the non-breeding season at more northern latitudes because they would be in better condition post-breeding.

Here, we study the northern flicker (*Colaptes auratus*), a woodpecker with partly reversed sex roles in which males do most of the excavation of nesting cavities [16], do more incubation and brooding of the young than females [17], feed the young slightly more [18] and provide care for a longer time [19]. In flickers, the costs of egg laying is low because not only are the eggs one of the smallest relative to body size of any bird, they also have one of the lowest energy contents of any bird species [20]. Although exact energy expenditure has not been quantified for the sexes, female flickers may buffer energetic costs of breeding by abandoning their offspring or reducing parental care during the post-fledging period, which is a common tactic in approximately 36% of females [19]. This suggests that many male flickers, which are left to care for offspring have less time to recoup breeding costs and may face greater energetic demands than females.

Male flickers have almost 100% assurance of paternity [21] meaning there would be little advantage for males to arrive at the breeding grounds early to acquire more extra-pair offspring. Thus, according to our new reframing of the fasting endurance hypothesis, because male flickers invest more time (and likely energy) into parental care they would be more likely to migrate farther than females to seek out the more benign southerly habitats. By contrast, the body size hypothesis predicts that females will migrate farther because males are slightly larger than females [16] and can withstand harsher conditions. The arrival time hypothesis also predicts males should winter farther north; although flickers do not defend feeding territories, males defend cavities [22] and are more philopatric to nest sites [23] than females. However, there is no evidence of different arrival timing between males and females on breeding sites [16,24]. Finally, although one sex does not appear to be dominant in flickers (E. A. Gow and K. L. Wiebe 2014, unpublished data), in several species of woodpeckers males are dominant over females [25,26], predicting that males may force females to move farther south (dominance hypothesis).

Currently, little is known about migration patterns of northern flickers. Northern populations are certainly migratory, whereas southern-breeding flickers appear to be sedentary [16] but this has not been thoroughly examined. Flickers are not currently a species of conservation concern, but the Breeding Bird Survey (BBS) shows that populations are declining at an average rate of −1.36% per year since 1966, across North America, with higher rates of decline in eastern compared with western North America [27]. Hence, in addition to examining sex-related differences in migration distance, we were interested in describing continent-wide movements of flickers, an ecologically valuable keystone species for cavity nesting and roosting animals [28], in order to better understand environmental factors which may be affecting the population dynamics of flickers across their geographical range.

## 3. Material and methods

We determined the autumn migration movements of northern flickers by using pooled data from both banding records and geolocators. The Canadian Bird Banding Office provided 1086 band recoveries for flickers throughout North America from 1915 to 2013 and we used 175 of these records from birds marked during breeding (15 May–31 July) and re-sighted/recovered during winter (15 October–1 March) to determine the movement of individual flickers from breeding to wintering sites. We eliminated those birds that were re-trapped within the same season (i.e. breeding or winter) because such movements are not migratory. We probably also excluded juvenile dispersal by excluding recoveries from the post-breeding autumn migratory period (1 August–14 October). There were 41 records from birds banded as part of a long-term study (1997–2013) at Riske Creek, British Columbia, Canada (52.03^°^ N, 122.515^°^ W). In addition, seven re-sighting records from Riske Creek that were not reported to the banding office were included. One bird that was banded at Riske Creek and recovered in Newfoundland after flying across the continent for 4835 km was excluded from analysis because it was not a typical migration. In summary, we used a combination of continent-wide band recovery data (*n*=142), band recoveries/re-sightings from a single population at Riske Creek (*n*=41) and geolocator locations from 17 flickers tagged at Riske Creek to map the wintering areas, migratory distances and general wintering patterns of flickers.

In 2010–2012, we attached geolocators to 51 male and 25 female flickers during breeding between 15 May and 15 July, 2010–2012 at Riske Creek, and thus had additional migration and wintering details from 17 individuals (16 male and one female). Return rates of birds with geolocators were similar to natural return rates for our study site (see details in [29]). Flickers were caught at the nest cavity by flushing them into a net placed over the hole and then banded with a United States Geological Survey aluminium band and a unique colour band combination, sexed and aged as yearling (SY) versus older (ASY) based on moult [30]. The geolocator models (MK12 in 2010, MK20AS in 2011 and MK10 in 2012 weighing 0.9, 1.0 and 1.6 g, respectively; British Antarctic Survey, Cambridge, UK) were less than 1% of the average body mass (157 g ± 0.2 s.e., *n*=2161). We attached the devices using a leg loop backpack harness [31] with 45 lb test braided nylon cord tied and glued.

Prior to deploying the geolocators, we placed them outdoors with a similar view of the horizon for at least 7 days. The pre-deployment data were used to calculate a light threshold level and sun elevation angle for sunrise and sunset transitions for the stationary geolocators. The corresponding sun altitude for the calibration period was −4.3^°^±0.36 s.d., *n*=18 and the light threshold level was 16. Because flickers roost in cavities throughout the year [29], adjustments were made to the light-level transitions in the morning and evening to more accurately reflect the true sunrise and sunset times (E. A. Gow 2014, unpublished data). This method produced a total location error of mean 94±167 km (s.d.) or mean 60±107 km (s.d.) in latitude and mean 71±142 km (s.d.) in longitude, as determined from birds at a known location on their breeding territory. To obtain wintering locations from birds with geolocators, we first averaged locations over a period of 5 days and if the bird then appeared to be stationary (i.e. differences less than 2 s.d. of the error across several 5 day intervals), we determined location by averaging all days in the stationary period.

Using a pooled dataset (unless otherwise indicated) of both the banding data from across North America (*n*=41 from Riske Creek, *n*=142 from elsewhere) and geolocator data from Riske Creek (*n*=17), we examined whether wintering latitude was influenced by the fixed effects of sex, age and breeding latitude, and breeding longitude using a generalized linear model (GLM) with a Gaussian distribution and identity link function. We initially included the interaction effects between breeding longitude and latitude with sex and age, but because none was significant we removed the interactions from the model to increase power. Additionally, because there were no significant effects of longitude on wintering latitude, we removed longitude from our model. All statistical analyses were conducted in R 3.03 [32], error is expressed as ± s.d., unless indicated otherwise. All statistical tests are two-tailed and significance is *p*≤0.05.

## 4. Results

**Figure 1.**
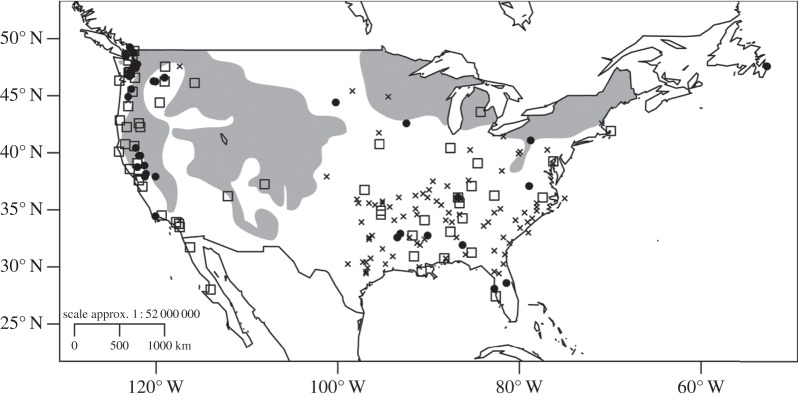
Location of wintering northern flickers by sex obtained from band recoveries and geolocator data. The shaded regions indicate average snowfall of more than 91.4 cm from 1961 to 1990 in the USA. Snowdepth was obtained from data from the National Oceanic and Atmospheric Administration's (NOAA) National Climatic Data Center (NCDC). Flickers tended to winter in regions with little snowfall and females wintered at significantly higher latitudes on average than males. Squares, male; filled circles, female; crosses, unknown.

**Figure 2.**
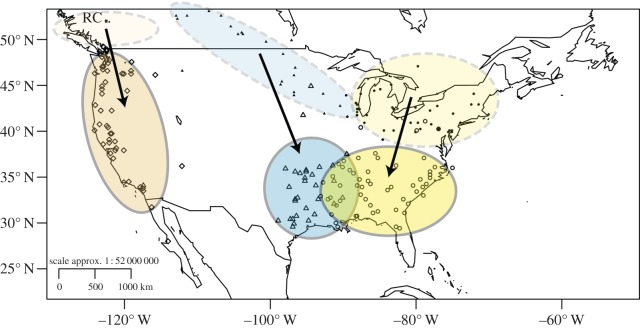
Connections between breeding and wintering location of flickers. Data are from northern flickers banded or carrying geolocators during the summer (between 15 May and 31 July) and recovered in winter (between 15 Oct and 1 March). Western populations (orange, circles) winter west of the Continental Divide, central populations (blue, triangles) winter in the south central USA and northeastern populations (yellow, squares) winter in the southeast. Breeding locations are indicated with filled symbols and non-breeding locations with open symbols. Riske Creek, the location of long-term banding and geolocator deployment is indicated by RC on the map. Polygons encompass approximately 90% of the points.

There were band recoveries from flickers that bred as far north as 53.08^°^ N and as far south as 27.25^°^ N. Of the 193 flickers banded in North America during the summer months, 79% moved more than 50 km from their breeding location and were probably migratory. Wintering locations of flickers were clustered along the west coast of the USA, and in other areas with little snow cover in the southeastern USA (figure 1]). Nearly all flickers breeding west of the Continental Divide, i.e. those from Riske Creek, plus one originating less than 100 km away, wintered along the west coast of North America from Vancouver, Canada, in the north to the Baja Peninsula, Mexico, in the south. Flickers breeding in the central region of the continent wintered in Texas, Oklahoma, Arkansas and Louisiana, whereas those breeding in the northeast wintered as far west as Arkansas and Louisiana and east to the Atlantic Ocean (figure 2]).

**Figure 3.**
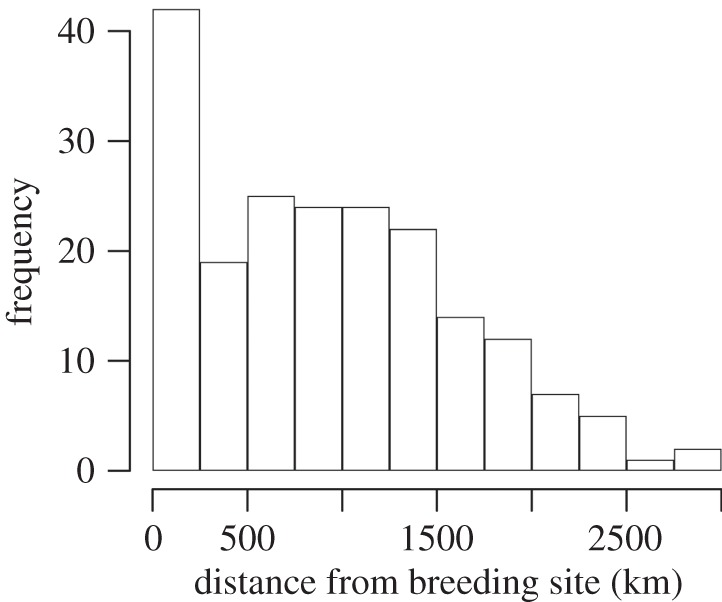
The distribution of distances travelled between breeding and wintering sites of individual northern flickers (*n*=199). Data are based on continent-wide banding recoveries and geolocator data.

There was considerable variation in the distance individuals moved (*mean*=913±701 km, *n*=197, range: 0–2882 km; figure 3). One male flicker with a geolocator travelled from Riske Creek to the Baja Peninsula, in Mexico a distance of approximately 2882 km, but migrations of more than 2000 km were typically rare (figure 3) and were only observed from the birds carrying geolocators. At the other extreme, there were four sedentary individuals, and three individuals that moved 200, 224 and 278 km from breeding to wintering sites in the eastern population (figure 2). Flickers that bred farther north also wintered farther north (GLM: *t*_72_=4.98, *p*<0.0001, estimate: 0.49±0.099 s.e., 0.68 (95% CI), and this pattern was consistent when non-migrants were removed (only individuals migrating more than 50 km; *r*=0.35, *p*<0.0001, *n*=160, estimate: 0.69±0.08). Because of a potential distance bias associated with the geolocator recovery method, we also analysed the data including only migratory birds from the banding recoveries and found that breeding latitude was positively correlated to migration distance (*r*=0.054, *p*=0.0053, *n*=139, estimate: 27.03±9.53).

**Figure 4.**
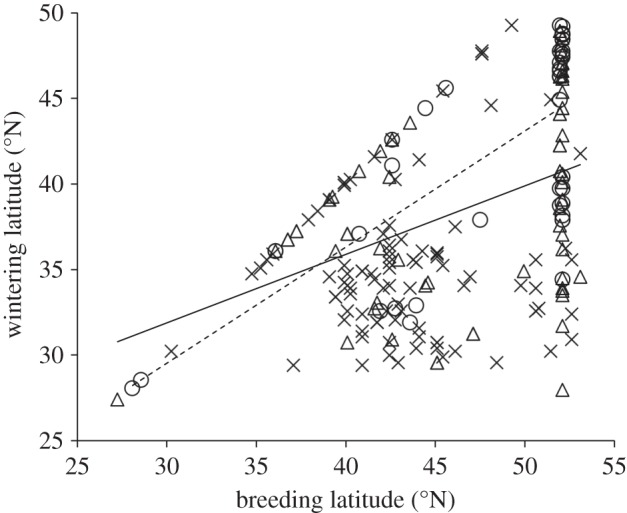
Comparison between breeding latitude and wintering latitude of northern flickers breeding at higher latitudes migrated farther distances as shown by 182 band recoveries from individuals banded between 15 May and 31 July, and then recaptured/recovered during the winter between 15 Oct to 1 March, and 17 flickers carrying geolocators. Females (dashed line) tended to winter closer to breeding sites, whereas males wintered farther from the breeding sites (solid line). Circles, female; triangles, male; crosses, unknown.

On the continental scale, females wintered significantly farther north than males (GLM: *t*_72_=−2.29, *p*=0.024, estimate: −2.62±1.17 s.e., −0.39 (95% CI); mean: male=38.68^°^±5.77^°^ N, female=41.76^°^±6.33^°^ N; figures 1 and 4), but there was no age effect (SY versus ASY birds: *t*_72_=1.13, *p*=0.26, estimate: 1.46±1.31 (s.e.), 4.07 (95% CI)). Because only some (*n*=75 out of 198) records included both age and sex, we also modelled each variable separately. Sex and breeding latitude remained significant predictors of wintering latitude (sex: *t*_102_=−2.55, *p*=0.012, estimate: −3.08±1.23 (s.e.), −0.71 (95% CI); breeding latitude: *t*_197_=8.07, *p*<0.001, estimate: 0.50±0.062 (s.e.), 0.62 (95% CI)), and age was not significant (*t*_154_=−0.32, *p*=0.75, estimate: −0.32±0.98 (s.e.), 0.75 (95% CI)). When only birds from Riske Creek were included, there were no age effects (GLM: *t*_54_=−0.01, *p*=0.99, estimate: 5.76±2.41 (s.e.), 10.49 (95% CI), and the sex-related pattern remained with females wintering farther north than males (*t*_54_=2.19, *p*=0.033, estimate: 0.37±2.21 (s.e.), 4.69 (95% CI); means: females: 990±923 km (s.d.), males 1200±665 km (s.d.); figure 1). Because of the potential distance bias from geolocator recoveries, we analysed only the band recovery data for migratory birds and found that male flickers migrated farther (*t*_65_=2.0, *p*=0.049) and wintered farther south than females (*t*-test: *t*_65_=−2.75, *p*=0.008).

## 5. Discussion

Banding and geolocator recoveries revealed several patterns in the migratory movements of northern flickers. At the continental scale, populations breeding in the north migrated farther than those breeding in the south. Additionally, populations west of the Rocky Mountains showed little overlap in wintering areas with populations breeding east of the Rockies. Most interesting, female flickers wintered farther north than males, which contradicts the pattern found in all other species of birds studied to date and is most consistent with the fasting endurance hypothesis.

At the species level, northern flickers are partial migrants, because some individuals in the south are non-migratory, but northern populations would be considered short-distance migrants as all individuals leave the breeding area. The location of wintering areas on the continent suggests the main attribute of suitable habitat is snow-free ground favourable for foraging (figure 1). Flickers feed almost exclusively on ground-dwelling ants during breeding [33] and such ground-dwelling insect prey are also the mainstay of the diet in the winter although more fruit may be incorporated [34,35]. Other climatic variables, such as rain, and cold temperatures are less likely than snow cover to restrict access to ground-dwelling arthropods. Our data show that migration in flickers is not leapfrog or chain migration and indicate that more northern populations must travel longer distances to reach locations that remain snow-free during winter creating an overlap in the wintering sites of both southern- and northern-breeding individuals. Among species with a large geographical breeding range, it is common for migration propensity to vary with latitude as we found in flickers [6]. Newton [1] suggests that at least 69 species of birds show similar latitude-based migration tendencies with northern populations tending to be more migratory and southern ones sedentary (e.g. red-tailed hawks (*Buteo jamaicensis* [36]) and European robins (*E. rubecula* [7]). More intensive studies of focal populations of flickers are needed, especially of those breeding in the south, to determine to what extent partial migration and residency exists within certain populations.

Migratory connectivity varied along the continuum between strong and weak. Flicker populations east and west of the Continental Divide did not overlap, which suggests the divide can act as a physical barrier to dispersal [37]. Flockhart & Wiebe [24] hypothesized that such a migratory divide keeps the red-and-yellow-shafted subspecies of flicker from introgressing. Studies of other North America species such as Swainson's thrushes (*Catharus ustulatus* [37]) and yellow warblers (*Dendroica petecha* [38]) have also found that populations do not cross the Continental Divide during migration which leads to segregation of western and eastern populations and thus migratory connectivity on the scale of the species entire geographical range. For flickers east of the Rocky Mountains, the central population and eastern population overlapped to a small extent on the wintering grounds pointing to moderate connectivity between the two populations. However, at a finer scale within a single breeding population of flickers in the west (i.e. Riske Creek), individuals wintered over a considerable latitudinal range suggesting weaker connectivity at the scale of a breeding population.

We found variation in migratory distances within the population at Riske Creek and among flickers at the continental scale, which suggests that decisions about the distance to travel are based on different costs and benefits to individuals. The variation was not based on age class but was related to sex, with males travelling farther south than females on average, although with considerable overlap in the wintering ranges of the sexes. Sex differences in migration distances are well documented in other bird species but all reported cases suggesting females migrate farther than males [7,12,15]. Support for the arrival time hypothesis in relation to sex has been found in European blackbirds [15] and in relation to age class in lesser black-backed gulls (*Larus fuscus* [39]). The dominance hypothesis has been supported for such animals as blue tits (*Cyanistes caeruleus* [40]), red-spotted newts (*Notophthalmus viridescens* [41]), red deer (*Cervus elaphus* [42]) and American dippers (*Cinclus mexicanus* [43]), and the body size hypothesis is supported in fishes (reviewed in [44]), manakins [45] and dark-eyed juncos (*Junco hyemalis* [12]). However, the pattern in flickers contradicts expectations from these three main hypotheses for differential migration. Instead, the more southern wintering locations of male flickers are most consistent with the fasting endurance hypothesis, assuming that their higher investment in care is physiologically more costly than that experienced by females. The recoveries of geolocators were male-biased and were associated with longer documented movements, so recovery methodology may have exaggerated the sex bias. However, methodology cannot entirely explain the sex bias in migration distance because when we removed the 17 geolocator birds, females still wintered farther north than males.

Reproductive differences may contribute to migratory tendency in a polygynous lekking species with female-only parental care (white-ruffed manakins, *Corapipo altera*; an altitudinal migrant) because males tended to be more migratory than females [45]. However, sex roles in reproduction were not examined in this species, instead it was suggested that migration differences could be attributed to the 14% larger body size in females [45]. In comparison, flicker males migrated farther than females, but body size differences are less than 3% [16], suggesting another factor drives the greater flight distances of male flickers. Here, we stress for the first time to our knowledge, that sex-biased investment in parental care may influence the optimal migration strategies of the sexes.

Other explanations for the sex differences in migration distance have been proposed but probably do not apply to flickers. For example, a difference in diet and habitat preference between the sexes such as is the case for hooded warblers (*Wilsonia citrina* [46]) may lead to a selection of different wintering habitats. However, there was no difference in the diet of male and female flickers at least during the breeding season [33]. Neither do the sexes have different habitat preferences when foraging [47]. If the fasting endurance hypothesis explains migratory distance in flickers, any additional energetic costs of flying further south among males must be outweighed by the increased access to food at the more benign southerly locations. As there is currently no data on energy expenditure during migration for flickers, this hypothesis should be tested by quantifying the physiological costs of flying different distances.

In conclusion, we documented strong migratory connectivity in flickers at the continental scale, with eastern and western populations divided. This division has probably had evolutionary significance by reducing hybridization between the eastern and western colour forms of flickers. It may also provide an explanation for the different rates of decline in the east and west (see BBS trend data [27]). We are the first to examine migration patterns in a species with partially reversed sex roles in parental care and showed that, in contrast to other studies, females wintered farther north than males. Hence, we propose that parental effort may be another factor that influences adaptive migratory strategies of individuals by affecting the physiological stress experienced by males versus females and encourage future studies that quantify energetic costs of parental care to more directly test this hypothesis.

## Data accessibility

The geolocator data are available from the Dryad Digital Repository: doi:10.5061/dryad.0r86b. Banding records can be obtained from the Canadian Bird Banding Office, Environment Canada. http://www.ec.gc.ca/bbo/default.asp?lang=En&n=D4C273DE-1. (Accessed 13 November 2014).
